# Probabilistic PARAFAC2

**DOI:** 10.3390/e26080697

**Published:** 2024-08-17

**Authors:** Philip J. H. Jørgensen, Søren F. Nielsen, Jesper L. Hinrich, Mikkel N. Schmidt, Kristoffer H. Madsen, Morten Mørup

**Affiliations:** 1 Department of Applied Mathematics and Computer Science, Technical University of Denmark, 2800 Kongens Lyngby, Denmarkjehi@dtu.dk (J.L.H.); mnsc@dtu.dk (M.N.S.); kristofferm@drcmr.dk (K.H.M.); 2 Danish Research Centre for Magnetic Resonance, Centre for Functional and Diagnostic Imaging and Research, Copenhagen University Hospital Amager and Hvidovre, 2650 Hvidovre, Denmark

**Keywords:** tensor decomposition, multi-way modeling, variational inference, orthogonality constraint, PARAFAC2

## Abstract

The Parallel Factor Analysis 2 (PARAFAC2) is a multimodal factor analysis model suitable for analyzing multi-way data when one of the modes has incomparable observation units, for example, because of differences in signal sampling or batch sizes. A fully probabilistic treatment of the PARAFAC2 is desirable to improve robustness to noise and provide a principled approach for determining the number of factors, but challenging because direct model fitting requires that factor loadings be decomposed into a shared matrix specifying how the components are consistently co-expressed across samples and sample-specific orthogonality-constrained component profiles. We develop two probabilistic formulations of the PARAFAC2 model along with variational Bayesian procedures for inference: In the first approach, the mean values of the factor loadings are orthogonal leading to closed form variational updates, and in the second, the factor loadings themselves are orthogonal using a matrix Von Mises–Fisher distribution. We contrast our probabilistic formulations to the conventional direct fitting algorithm based on maximum likelihood on synthetic data and real fluorescence spectroscopy and gas chromatography–mass spectrometry data showing that the probabilistic formulations are more robust to noise and model order misspecification. The probabilistic PARAFAC2, thus, forms a promising framework for modeling multi-way data accounting for uncertainty.

## 1. Introduction

Tensor decompositions are multi-way generalizations of matrix decompositions such as principal component analysis (PCA): A matrix is a second-order array with two modes, rows and columns, while a data cube is a third order array with the third mode referred to as slabs. When multi-way data have an inherent multi-linear structure, the advantage of tensor decomposition methods is that they capture this intrinsic information and often provide a unique representation without needing further constraints such as sparsity or statistical independence.

Applications of tensor factorization originated within the field of psychometrics [1,2] and have been widely useful in other fields such as chemometrics [3], for example, to model the relationship between excitation and emission spectra of samples of different mixed compounds obtained by fluorescence spectroscopy [4]. Tensor decomposition is today encountered in practically all fields of research including signal processing, neuroimaging, and information retrieval (see also [5,6,7]).

The two most prominent tensor decomposition methods are (i) the Tucker model [8], where the so-called core array accounts for all multi-linear interactions between the components of each mode, and (ii) the CandeComp/PARAFAC (CP) model [1,2,9], where interactions are restricted to be between components of identical indices across modes, corresponding to a Tucker model with a diagonal core array. Both models can be considered generalizations of PCA to higher-order arrays, with the Tucker model being more flexible at the expense of reduced interpretability. The CP model has been widely used primarily due to its ease of interpretation and its uniqueness [6,10].

In the CP model, the components are assumed identical across measurements, varying only in their scaling. In many situations, this is too restrictive—for example, when the number of samples vary across a mode. Furthermore, violation of the CP structure within chemometrics can be caused by retention time shifts [11,12], whereas in neuroimaging, such violations may be induced by subject and trial variability [6] invalidating the use of the CP model. To handle variability while preserving the uniqueness of the representation, the Parallel Factor Analysis 2 (PARAFAC2) model was proposed [2]. It admits individual loading matrices for each entry in a mode while preserving uniqueness properties of the decomposition by imposing consistency of the Gram matrix (i.e., the loading matrix left multiplied by its transpose, thereby imposing consistency in how components are co-expressed across samples) [13,14,15]. It has since been applied within diverse application domains, including handling variations in elution profiles due to retention shifts in chromatography [11]; monitoring and fault detection facing unequal batch lengths in chemical processes [16]; in neuroimaging to analyze latency changes in frequency resolved evoked EEG potentials [17], to extract common connectivity profiles in multi-subject fMRI data accounting for individual variability [18], and to characterize dynamic functional connectivity [19]; for cross-language information retrieval [20]; as well as for music and image tagging [21,22]. Recently, efforts have been made to scale the PARAFAC2 model to large-scale data [23,24,25], enhance the robustness and efficiency of the conventional direct fitting algorithm [26,27], and apply a non-negativity constraint also on the varying mode [28,29] as well as broader sets of constraints based on alternating directions of the method of multipliers [30].

Traditionally, tensor decompositions have been based on maximum likelihood inference using alternating least squares estimation in which the components of a mode are estimated while keeping the components of other modes fixed. Initial probabilistic approaches defined probability distributions over the component matrices and the core array but relied on maximum likelihood estimates for determining a solution [31,32]. However, the Bayesian approach presented here makes inference with respect to the posterior distributions of the model parameters and can thus be used to assess uncertainty in the parameters and noise estimates. Most work on probabilistic tensor decomposition has focused on the TUCKER and CP models using either Markov Chain Monte Carlo (MCMC) sampling [33,34,35] or variational inference [36,37,38,39]. The CP and Tucker models have been extended to model sparsity [35,40,41], non-negativity [42], and non-linearity [33,43] in component loadings. Heteroscedastic noise modeling has been discussed in the context of the CP model [41,44,45] and Tucker model [46], the latter also providing a generalization of tensor decomposition to exponential family distributions. A review and toolbox for probabilistic tensor decompositions are given in [45]. For component matrices with orthogonal components, recent work has explored using the von Mises–Fisher Matrix (vMF) distribution in the CP model [47] and the block-term decomposition model defined as a sum of Tucker models [48]. The former used a MAP-based estimation—which is not a fully Bayesian approach—and the latter used a variational Bayesian inference approach. In addition to using a variational Bayesian inference approach to the vMF distribution, we also explore another orthogonal formulation that is applicable beyond the PARAFAC2 structure.

Benefits of probabilistic modeling include the ability to account for uncertainty and noise while providing tools for model order selection. Whereas probabilistic modeling can be directly applied to the CP and TUCKER models extending probabilistic PCA [49], a probabilistic treatment of the PARAFAC2 model faces the following two key challenges:

(i) The ability to impose orthogonality on variational factors (necessary for imposing the PARAFAC2 structure).
(ii) Handling the coupling of these orthogonal components.

In this paper, we address these two challenges and derive the probabilistic PARAFAC2 model. In particular, we investigate two different formulations of the orthogonality constraint and demonstrate how the orthogonality of variational factors as in the least squares estimation for conventional PARAFAC2 can be obtained in closed form using the singular value decomposition. We exploit how the probabilistic framework admits model order quantification by the evaluation of model evidence and automatic relevance determination. We contrast our probabilistic formulation to conventional maximum likelihood estimation on synthetic data as well as fluorescence spectroscopy and gas chromatography–mass spectrometry data, highlighting the utility of the probabilistic formulation facing noise and model order misspecification (A short workshop contribution in brief presenting the proposed probabilistic PARAFAC2 was presented in [50]).

## 2. Methods

The three-way CP model can be formulated as a series of coupled matrix decompositions,

$$X_{k}=AD_{k}F^{⊤}+E_{k},$$

where $X_{k}\in R^{I\times J}$ is the *k*’th slab of the three-way array X with dimensions I×J×K. Let *M* be the number of components in the model; then, the matrix A with dimensions I×M contains the loadings for the first mode and F with dimensions J×M contains the loadings for the second mode. The matrices $D_{k}$, k=1,…,K, are diagonal with dimensions M×M and contain the loadings for the third mode. These are usually written as a single matrix $C\in R^{K\times M}$, where the *k*’th row contains the diagonal of $D_{k}$. $E_{k}$ denotes the residuals for the *k*’th slab with dimensions I×J. Notice that the structure of the first and second mode are invariant across the third mode in this model.

The PARAFAC2 model extends the CP structure by letting a mode have individual factors $F_{k}$ for each slab. The extension allows for a varying number of observations in the chosen mode. This model would be as flexible as PCA on the concatenated data $\left[X_{1},X_{2},\ldots X_{K}\right]$ if not for the additional constraint that each Gram matrix of $F_{k}$ be identical, $F_{k}^{⊤}F_{k}=\Psi$, which is a necessary constraint in order to obtain unique solutions [51]. The three-way PARAFAC2 model can thus be written as

$$X_{k}=AD_{k}F_{k}^{⊤}+E_{k}\;s.t.\;F_{k}^{⊤}F_{k}=\Psi .$$

Modeling Ψ explicitly can be difficult, but it is necessary and sufficient [15] to have $F_{k}=P_{k}F$, with $P_{k}$ being a columnwise orthogonal J×M matrix and F a M×M matrix; thus, the model can be written as

(1) $$X_{k}=AD_{k}F^{⊤}P_{k}^{⊤}+E_{k}\;s.t.\;P_{k}^{⊤}P_{k}=I.$$

In the following, we describe the conventional direct fitting algorithm [15] for parameter estimation in the PARAFAC2 model before we introduce the probabilistic model formulation in Section 2.3.

### 2.1. Direct Fitting Algorithm

The parameters in the PARAFAC2 model in (1) can be estimated using the alternating least squares algorithm [15], minimizing the constrained least squares objective function,

$$\underset{A,F,\left\{P_{k},D_{k}\right\}\;\forall_{k}}{arg min}\sum_{k}{∥X_{k}-{AD}_{k}F^{⊤}P_{k}^{⊤}∥}^{2}\;s.t.\;P_{k}^{⊤}P_{k}=I.$$

For fixed A, $D_{k}$, and F, the $P_{k}$ that minimizes the *k*’th term in the objective function is equal to

(2) $$\underset{P_{k}}{arg max}\mathrm{Tr}\left(FD_{k}A^{\;⊤}X_{k}P_{k}\right)$$

and can be computed as [15,52]

(3) $$P_{k}=V_{k}U_{k}^{⊤}$$

where $V_{k}$ and $U_{k}$ come from the singular value decomposition (SVD) decomposition

$$U_{k}S_{k}V_{k}^{⊤}=FD_{k}A^{\;⊤}X_{k}.$$

Upon fitting $P_{k}$, each slab $X_{k}$ of the tensor can be projected onto $P_{k}$, thereby leaving the remaining parameters to be fitted as a CP model minimizing

(4) $$\underset{A,F,\{D_{k}\}}{arg min}\sum_{k}{∥X_{k}P_{k}-{AD}_{k}F^{⊤}∥}^{2}.$$

A solution to (4) is well explained by Bro in [3]. A well-known issue with maximum likelihood methods is that it can lead to overfitting due to noise and a lack of uncertainty in the model parameters, resulting in robustness issues, which we attempt to provide a solution for by advancing the PARAFAC2 model to a fully Bayesian setting.

#### Model Selection

A general problem for latent variable methods is how to choose the model order, *M*. A popular heuristic can be formed by how well the model fits the data given as

(5) $$R2=1-\frac{\sum_{k}{∥X_{k}-{AD}_{k}F^{⊤}P_{k}^{⊤}∥}^{2}}{\sum_{k}{∥X_{k}∥}^{2}}.$$

However, this measure will simply increase until the model incorporates enough parameters to completely fit the data, thus eventually leading to overfitting. The model selection criterion would only be based on the expected noise level.

Another popular heuristic is the core consistency diagnostic (CCD), originally developed for the CP model [53], but that has shown useful for the PARAFAC2 model as well [54]. It is based on the observation that the CP model can be seen as a constrained Tucker model, where the core array is enforced to be a superdiagonal array of ones. The principle behind CCD is to measure how much the CP model violates this assumption of a superdiagonal core array of ones by re-estimating the core array of the CP model to fit the Tucker model, denoted G, while keeping the loadings fixed and then calculating the CCD according to

$$\mathrm{CCD}=100\left(1-\frac{||G-{I||}_{F}^{2}}{{\left||I|\right|}_{F}^{2}}\right),$$

in which I is the superdiagonal core array and $\left|\right|\cdot {\left|\right|}_{F}$ denotes the Frobenius norm. The PARAFAC2 model can be written as a CP model for each slab as in (4); thus, the core array can be estimated in the same way as for the standard CP model. This approach was evaluated on synthetic as well as real data sets by [54], where the conclusion was that even though the CCD can be seen as an useful parameter for determining model order, it is not recommended in practice without considering other diagnostic measures, including inspecting the residuals and the loadings.

### 2.2. Variational Bayesian Inference

In Bayesian modeling, the posterior distribution of the parameters θ is computed by conditioning on the observed data X using Bayes’ rule, p(θ|X)=p(X|θ)p(θ)/p(X). The posterior is thereby given as the product of the likelihood p(X|θ) and the prior probability of the parameters p(θ) divided by the probability of the observed data p(X) under the model, also known as the marginal likelihood. Evaluating the marginal likelihood is, in general, intractable; instead, a variational approximation can be found by fitting a distribution q(θ)—called the variational distribution—to the posterior [55] minimizing the Kullback–Leibler (KL) divergence, given by

$$q^{★}\left(\theta \right)=\arg\;\min\;\mathrm{KL}\left[q(\theta )∥p(\theta |X)\right].$$

Minimizing the KL divergence is solved by maximizing a related quantity, the evidence lower bound (ELBO).

ELBO(q(θ))=E[logp(θ,X)]−E[logq(θ)].

A common choice is a variational distribution that factorizes over the parameters, known as a mean-field approximation, $q\left(\theta \right)=\prod_{j}q_{j}\left(\theta_{j}\right)$. Note that, for convenience, we choose distributions belonging to the exponential family, as this allows closed-form solutions to be found. The optimal variational distribution can then be found by iterative updates of the form

(6) $$q_{j}\left(\theta_{j}\right)\propto \exp\left(E_{-j}\left[\log p\left(\theta_{j},\theta_{-j},X\right)\right]\right),$$

where $E_{-j}\left[\cdot \right]$ denotes the expectation over the variational distribution except $q_{j}$. For a comprehensive overview of variational inference, see for example [56,57], and for Bayesian inference in general, see [58].

### 2.3. Probabilistic PARAFAC2

We propose two probabilistic PARAFAC2 variants using the formulation in (1), which differ only in how the orthogonality of $P_{k}$ is handled. The constraint $P_{k}^{⊤}P_{k}=I_{M}$ has the probabilistic interpretation that $E\left[P_{k}^{⊤}P_{k}\right]=I_{M}$, in which the $P_{k}$ is an orthogonal matrix, which we call model (i). Another interpretation is to enforce that the expected value $E[P_{k}]$ is an orthogonal matrix and implies $E{\left[P_{k}\right]}^{⊤}E\left[P_{k}\right]=I_{M}$—which we call model (ii). The main motivation for the latter approach being the interpretation of the orthogonal factor is identical to that of the maximum likelihood estimation. However, the resulting components are no longer themselves restricted to the set of orthogonal matrices, namely, the Stiefel manifold. As such, the model (ii) becomes more flexible as only the mean parameters of the variational approximation are constrained to be orthogonal and not the expectation of their inner product, as required for every realization of the underlying distribution to conform to the PARAFAC2 model. We include the latter model formulation, as it provides simple closed-form updates similar to the conventional direct fitting PARAFAC2 algorithm, as shown below. The updates for (ii) are derived by constraining the mean of a matrix normal (MN) distribution within the variational approximation to the Stiefel manifold, whereas the model (i) formulation is based on [59] and uses a matrix von Mises–Fisher (vMF) Matrix distribution, which only has support on the Stiefel manifold. We accordingly present the following two generative models, (i) and (ii), for the probabilistic PARAFAC2:

$$\begin{array}{cccc} \; & \; & a_{i\cdot } & ∼N(0,I_{M})\\ \; & \; & f_{m\cdot } & ∼N(0,I_{M})\\ \; & \; & c_{k\cdot } & ∼N(0,\mathrm{diag}\left(\alpha^{-1}\right))\\ \left(i\right) & \; & P_{k} & ∼\mathrm{vMF}\left(0\right)\\ \left(\mathrm{ii}\right) & \; & P_{k} & ∼\mathrm{MN}\left(0,I_{J},I_{M}\right)\;s.t.\;E{\left[P_{k}\right]}^{⊤}E\left[P_{k}\right]=I_{M}\\ \; & \; & \tau_{k} & ∼\;\mathrm{Gamma}(a_{\tau_{k}},b_{\tau_{k}})\\ \; & \; & X_{k} & ∼N(AD_{k}F^{⊤}P_{k}^{⊤},\tau_{k}^{-1}I_{J}), \end{array}$$

where $a_{i\cdot }$ denotes the *i*th row of the matrix A and similarly for $f_{m\cdot }$ and $c_{k\cdot }$. We denote the set of all ${\left\{P_{k}\right\}}_{k=1,2,\ldots ,K}$ as P. For the rate-scale Gamma distribution, the hyper-parameters $a_{\tau_{k}}$ and $b_{\tau_{k}}$ are user defined. α defines the length scale of each component and can thus be used for automatic relevance determination (ARD) by turning off excess components by concentrating their distributions at zero when $\alpha_{m}$ is large [56]. In this paper, we use the MAP estimate of $\alpha_{m}$ as we are more interested in the model’s pruning ability than uncertainty on $\alpha_{m}$. Pruning excess components is a challenging task, see [45] for ARD within Bayesian inference in the CP and Tucker models, and [60] for Bayesian shrinkage priors in general. Lastly, we allow the noise $\tau_{k}$ to vary across slabs, thereby accounting for potential different levels of the noise (i.e., assuming heteroscedastic noise) across slabs.

### 2.4. Variational Update Rules

The inference is based on the following factorized distribution,

$$q\left(\theta \right)=q\left(A\right)q\left(C\right)\prod_{m}q\left(f_{m\cdot }\right)\prod_{k}q\left(P_{k}\right)q\left(\tau_{k}\right)$$

leading to the following ELBO,

(7) $$\begin{array}{cc} \mathrm{ELBO}(q(\theta ))= & E[\log p(X,\theta )]-E[\log q(\theta )]\\ = & E[\log p(X|A,C,F,P,\tau )]+E[\log p(A)]\\ \; & +E[\log p(C|\alpha )]+E[\log p(F)]\\ \; & +E[\log p(P)]+E[\log p(\tau )]\\ \; & +h(q(A))+h(q(C))+h(q(F))\\ \; & +h(q(P))+h(q(\tau )). \end{array}$$

Expanding the variational factors, as given by (6), the resulting variational distributions and update rules are given in Table 1. The update for the factor matrix F is non-trivial, and to obtain a closed-form solution we employ a componentwise updating scheme inspired by the non-negative matrix factorization literature [61,62,63]. For each latent parameter, we use (6) and moment matching to determine the optimal variational distributions.

These updates rules are used for implementing a computational algorithm for probabilistic PARAFAC2, where each factor A,C,F,P, and τ is updated conditionally on all other factors. This leads to an alternating optimization algorithm that, given an initial solution (randomized or starting from the MAP solution), iteratively maximizes the evidence lower bound, Equation (7), until the relative change in ELBO is below a convergence criteria or a maximum number of iterations is reached. Finding the optimal solution is a non-convex optimization problem that is sensitive to initialization and the order of the updates.

#### 2.4.1. Von Mises–Fisher Loading

In the von Mises–Fisher model for the loading $P_{k}$, the variational distribution is given by

$$\mathrm{vMF}\left(P_{k}|B_{P_{k}}\right)=\kappa {\left(J,B_{P_{k}}^{⊤}B_{P_{k}}\right)}^{-1}\exp\left(\mathrm{tr}[B_{P_{k}}^{⊤}P_{k}]\right),$$

which is defined on the Stiefel manifold, $P_{k}^{⊤}P_{k}=I$. The normalization constant is given by κ=F1012J,14BPk⊤BPkvJ,M, where $v_{J,M}$ is the volume of the *J*-dimensional Stiefel manifold described by *M* components [64]. The hypergeometric function with matrix argument F10·,· can be calculated more efficiently using the SVD of $B_{P_{k}}=U_{k}S_{k}V_{k}^{⊤}$, since F1012J,14BPk⊤BPk=F1012J,14Sk2 [64].

Computing expectations over the vMF matrix distribution requires evaluating the hypergeometric function and can be performed as described by [59]. (^†^ Source code for approximating the hypergeometric function is available online http://staff.utia.cz/smidl/files/mat/OVPCA.zip (accessed on 28 February 2017). This code was used with default settings and without modifications in the experiments. We also share it with the probabilistic PARAFAC2 code at https://github.com/philipjhj/VBParafac2 (accessed on 28 February 2017)). Note that it follows from the vMF matrix distribution that $E\left[P_{k}^{⊤}P_{k}\right]=I$, but in general $E{\left[P_{k}\right]}^{⊤}E\left[P_{k}\right]\neq I$. However, if an orthogonal summary representation is desired, one can inspect the mode of the vMF given by $U_{k}V_{k}^{⊤}$.

#### 2.4.2. Constrained Matrix Normal Loading

In the constrained matrix normal (cMN) model for the variational factor of the loadings $P_{k}$, we consider the distribution

$$\begin{array}{cc} c\mathrm{MN}(P_{k}|M_{P_{k}},I_{J},\Sigma_{P_{k}})= & \frac{\exp\left\{-\frac{1}{2}\mathrm{trace}\left(\Sigma_{P_{k}}^{-1}{\left(P_{k}-M_{P_{k}}\right)}^{⊤}I_{J}^{-1}\left(P_{k}-M_{P_{k}}\right)\right)\right\}}{{\left(2\pi \right)}^{IM/2}|\Sigma_{P_{k}}{|}^{I/2}{\left|I_{J}\right|}^{M/2}},\\ \; & s.t.\;M_{P_{k}}^{⊤}M_{P_{k}}=I. \end{array}$$

Instead of using the free form variational approach, we maximize (7) as a function of the mean parameter $M_{P_{k}}$ subject to the orthogonality constraint $M_{P_{k}}M_{P_{k}}^{⊤}=I_{M}.$

The constraint consequently causes (7) to be constant except for the linear term of the expected log of the probability density function of the data. The reason for this is that all other terms do not depend on $M_{P_{k}}$ or only on the matrix product $M_{P_{k}}M_{P_{k}}^{⊤}$, which is equivalent to the identity matrix, resulting in the optimization problem

$$\begin{array}{c} \underset{M_{P_{k}}}{\arg\;\max}\;\mathrm{ELBO}\left(M_{P_{k}}\right)\;s.\;t.\;M_{P_{k}}{M_{P_{k}}}^{⊤}=I \end{array}$$

where

$$\begin{array}{c} \mathrm{ELBO}\left(M_{P_{k}}\right)=\sum_{k}E\left[\tau_{k}\right]\mathrm{Tr}\left(E\left[F\right]E\left[D_{k}\right]E\left[A^{⊤}\right]X_{k}M_{P_{k}}\right)+c. \end{array}$$

This is equal to (2) except for a scalar leading to the same solution as for the maximum likelihood estimation method, as given in (3). Detailed derivations of the expression above are given in the Appendix A and Appendix B. The variance parameter $\Sigma_{P_{k}}$ in the variational distribution follows from moment matching using (6).

#### 2.4.3. The **F** Matrix

The updates for $f_{m\cdot }$ are non-trivial due to an inter-component dependency. The quadratic term in (6) for F is

$$\begin{array}{l} E_{-F}\left[a_{i\cdot }D_{k}F^{⊤}{P_{k}}^{⊤}P_{k}FD_{k}{a_{i\cdot }}^{⊤}\right]\\ \;=E_{-F}\left[\mathrm{Tr}\left(FD_{k}{a_{i\cdot }}^{⊤}a_{i\cdot }D_{k}F^{⊤}{P_{k}}^{⊤}P_{k}\right)\right]\\ \;=\mathrm{Tr}(FE_{-F}\left[D_{k}{a_{i\cdot }}^{⊤}a_{i\cdot }D_{k}\right]F^{⊤}E_{-F}\left[{P_{k}}^{⊤}P_{k}\right])\\ \;=\sum_{mm^{\prime }}{\left(FE\left[D_{k}{a_{i\cdot }}^{⊤}a_{i\cdot }D_{k}\right]F^{⊤}\right)}_{mm^{\prime }}{\left(E\left[{P_{k}}^{⊤}P_{k}\right]\right)}_{mm^{\prime }}\\ \;=\sum_{mm^{\prime }}f_{m\cdot }E\left[D_{k}{a_{i\cdot }}^{⊤}a_{i\cdot }D_{k}\right]f_{m^{\prime }\cdot }^{T}E\left[p_{\cdot mk}^{T}p_{\cdot m^{\prime }k}\right]\\ \;=\sum_{m}f_{m\cdot }E\left[D_{k}{a_{i\cdot }}^{⊤}a_{i\cdot }D_{k}\right]E\left[p_{\cdot mk}^{T}p_{\cdot mk}\right]f_{m\cdot }^{T}\\ \;+2\sum_{m}\sum_{m^{\prime }\setminus m}f_{m\cdot }E\left[D_{k}{a_{i\cdot }}^{⊤}a_{i\cdot }D_{k}\right]E\left[p_{\cdot mk}^{T}p_{\cdot m^{\prime }k}\right]f_{m^{\prime }\cdot }^{T}, \end{array}$$

where we see that the quadratic term separates into a quadratic and linear part, revealing the linear inter-component dependency.

#### 2.4.4. Non-Trivial Expectations

An overview of all the factors and their updates are given in Table 1. Below, we detail some non-trivial expectations and the necessary steps to compute them. The first group of expectations deals with having the diagonal matrix $D_{k}$ left and right multiplied with an inner term. The first case is the following expectation,

$$\begin{array}{c} E[D_{k}{a_{i\cdot }}^{⊤}a_{i\cdot }D_{k}] \end{array}$$

which is equivalent to the Hadamard product of the outer product of the diagonal of the surrounding matrix with itself and the inner matrix; so, we can separate the expectation into two parts

$$\begin{array}{cc} E[D_{k}{a_{i\cdot }}^{⊤}a_{i\cdot }D_{k}]= & E\left[{c_{k\cdot }}^{⊤}c_{k\cdot }\right]\circ E\left[{a_{i\cdot }}^{⊤}a_{i\cdot }\right], \end{array}$$

where $c_{k\cdot }$ is the vector containing the diagonal elements of $D_{k}$. The same rule applies for the following expectation:

$$\begin{array}{cc} E[D_{k}F^{⊤}{P_{k}}^{⊤}P_{k}FD_{k}]= & E\left[{c_{k\cdot }}^{⊤}c_{k\cdot }\right]\circ E\left[F^{⊤}{P_{k}}^{⊤}P_{k}F\right], \end{array}$$

where the second expectation becomes trivial when using the vMF prior (ii) as the matrix product ${P_{k}}^{⊤}P_{k}$ is the identity matrix. However, when using the matrix normal distribution (i), we obtain

$$\begin{array}{cc} E[{P_{k}}^{⊤}P_{k}]= & \mathrm{Tr}\left(\Sigma_{P_{k}}\right)+I_{M}, \end{array}$$

which leads to the element with index ij of the expectation to be equal to

$$\begin{array}{cc} E{\left[F^{⊤}{P_{k}}^{⊤}P_{k}F\right]}_{ij} & =E[\sum_{m}{\left(F^{⊤}\right)}_{im}{\left({P_{k}}^{⊤}P_{k}F\right)}_{mj}]\\ \; & =E[\sum_{m}F_{mi}^{⊤}\sum_{m^{\prime }}{\left({P_{k}}^{⊤}P_{k}\right)}_{mm^{\prime }}F_{m^{\prime }j}]\\ \; & =\sum_{m}\sum_{m^{\prime }}E\left[F_{mi}^{⊤}F_{m^{\prime }j}\right]E\left[{\left({P_{k}}^{⊤}P_{k}\right)}_{mm^{\prime }}\right]. \end{array}$$

Since the *m*’th and $m^{\prime }$ components are independent, we have

$$\begin{array}{cc} \; & E[F_{mi}^{⊤}F_{m^{\prime }j}]=\\ \; & \;\left\{\begin{array}{cc} E\left[F_{mi}^{⊤}\right]E\left[F_{m^{\prime }j}\right]+{\left(\Sigma_{f_{m\cdot }}\right)}_{ij} & \mathrm{for}\;m=m^{\prime }.\\ E\left[F_{mi}^{⊤}\right]E\left[F_{m^{\prime }j}\right] & \mathrm{for}\;m\neq m^{\prime }. \end{array}\right. \end{array}$$

These are the most involved expectations when computing the update rules, and the remaining are either simpler or depend upon the expectations derived here.

### 2.5. Noise Modeling

The probabilistic formulation of PARAFAC2 requires the specification and estimation of the noise precision τ. We presently consider two specifications, i.e., homoscedastic noise in which the noise of each slab $X_{k}$ is identical—i.e., $\tau_{1}=\ldots =\tau_{K}$—as assumed in the direct fitting algorithm, and heteroscedastic noise, where the model includes a separate precision for each of the *K* slabs.

### 2.6. Model Selection

A benefit of a fully probabilistic formulation of the PARAFAC2 model is that it provides model order quantification using tools from Bayesian inference, see [45,60], respectively, for details in the context of probabilistic tensor models and Bayesian inference in general. We presently exploit automatic relevance determination by learning the length scale α, see also [56]. In practice, we use the MAP estimates for the automatic relevance determination because we are more interested in the pruning ability than the uncertainty estimates on α. If desired, a variational estimate is easily found by letting $\alpha_{m}$ follow a Gamma distribution, c.f. [49]. Finally, the estimated ELBO on the data can also be used to compare different model orders.

### 2.7. Computational Complexity

The computational complexity of probabilistic PARAFAC2—for a third-order tensor $X\in R^{I\times J\times K}$ with *M* components—is the same as its maximum likelihood alternative, namely, $O(I\cdot J\cdot K\cdot M+K\cdot M^{3})$ where the first term stems from the matricized tensor Khatri–Rao product (MTTKRP) and the second from the inversion (or SVD) of an M×M matrix in connection with updating $P_{k}$ for k=1,2,…,K. The MTTKRP cannot be avoided, but caching of the sufficient statistics can make resulting calculations more efficient, although the computational complexity remains unchanged. Usually, I,J, and *K* are much greater than *M*; so, the MTTKRP becomes the limiting factor. Importantly, a limitation of the variational Bayesian formulation of PARAFAC2 is that one cannot directly use the projection trick of PARAFAC2, where the *K* mode is projected such that $X_{k}P_{k}=Y_{k}$, and Y then has a PARAFAC structure. The trick relies on $P_{k}$ being orthogonal, but in the variational formulation, the expectation of $E[P_{k}]$ is used instead of $P_{k}$; thus, it is no longer exactly orthogonal. A remedy to this is either using sampling or a maximum a priori estimate for which $P_{k}$ is exactly orthogonal, although neither approach changes the computational complexity of probabilistic PARAFAC2.

## 3. Results and Discussion

We evaluate the proposed models on both synthetic data and three real data sets: an amino acid fluorescence (AAF) data set and two gas chromatography–mass spectrometry (GC-MS) data sets. For comparison, we include the least squares PARAFAC2 direct-fit (Direct Fit) [15], probabilistic CP with normal distributed factors and a Gamma ARD-prior with either homoscedastic (VB PARAFAC Δ) or heteroscedastic (VB Parafac Ω) noise modeling, probabilistic Tucker (VB TUCKER) [48], and Bayesian relaxed matrix factorization (rMFT) [65]. For the proposed probabilistic PARAFAC2 methods, we initialize the model parameters as the PARAFAC2 solution computed using the direct fitting algorithm (as implemented by Bro [15] at http://www.models.life.ku.dk/go?filename=parafac2.m (accessed on 13 October 2017)) and repeat the initialization five times for the synthetic data and 50 times for the real data to minimize the risk of getting stuck in a local extrema. The final model parameters are chosen as the parameters with the lowest R2 for the direct fitting models and the highest ELBO for the probabilistic models among the fitted models. Each model estimation is limited to $10^{4}$ iterations for the synthetic data and $5\times 10^{4}$ iterations for the real data. If the relative improvement in R2 for the direct fitting models and the ELBO for the probabilistic models after an iteration goes below $10^{-9}$, we invoke an early stop. Empirically, we experienced better learning of the probabilistic models by keeping the precision parameter of the added noise fixed for some number of iterations while estimating the length scale α. We choose this delay to last for the first 50 iterations. The hyper-parameters of the precision were set to (shape, scale) = $\left(a_{\tau_{k}},b_{\tau_{k}}\right)=\left(1,10^{32}\right)$ in order to be uninformative for the variational distribution, as their influence on the updated parameters is very small on the considered data sets.

### 3.1. Synthetic Data

To investigate the performance of the proposed model, we generate synthetic data sets in a similar manner as in [15]. We generated the data tensor X by sampling A from a zero-mean isotropic multivariate normal distribution with unit variance. F was taken from a Cholesky factorization of an M×M matrix with 1’s in its diagonal and 0.4 in all the off-diagonal elements. This essentially keeps the *M* components from being too similar. Each element of C was sampled from a uniform distribution on the interval 0 to 30. $P_{k}$ was constructed by the standard orthonormalization function in MATLAB of a set of vectors sampled from a zero-mean isotropic multivariate normal distribution with unit variance. The synthetic data sets were generated with either homoscedastic or heteroscedastic additive noise at different signal-to-noise ratios (SNR) in the interval [−20,10] dB, with increments of 2 dB. Each configuration was generated 10 times, resulting in 320 data sets. Each data set was given the dimensions 50×50×10 with M=4 components.

**Figure 1:**
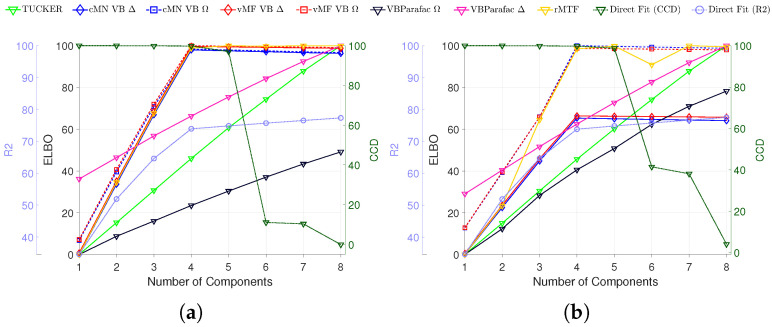
Mean of model selection criteria R2 and CCD reported on the conventional PARAFAC2, and the ELBO for the TUCKER, rMTF, probabilistic PARAFAC, and probabilistic PARAFAC2 models, with 1 to 8 components on 10 synthetic data sets with added homoscedastic (**a**) and heteroscedastic (**b**) noise both with an SNR equal to 4. To make the results comparable, all ELBO values for each criterion and model (but across noise model types) have been normalized to be in the range of 0 to 100. In the legend, Δ indicates a homoscedastic noise model and Ω indicates a heteroscedastic noise model.

**Figure 2:**
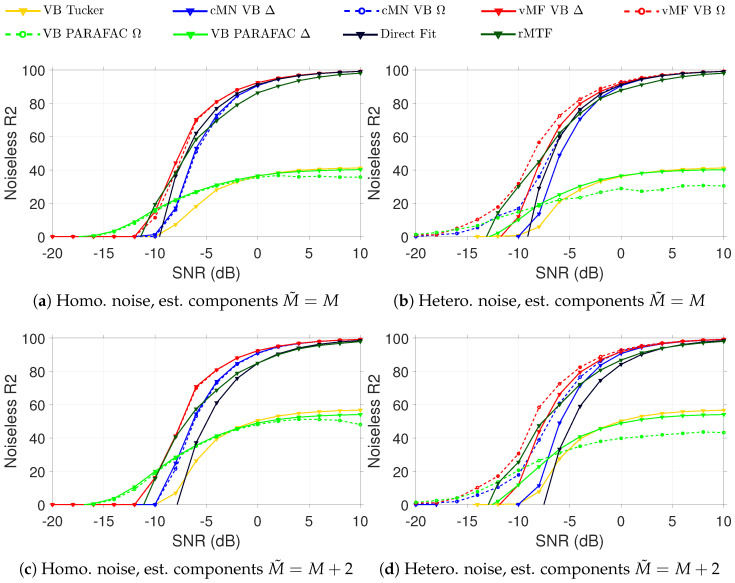
Recovery of the underlying signal in synthetic data with varying levels of homoscedastic (**a**,**c**) and heteroscedastic (**b**,**d**) added noise, as measured by noiseless R2. For the conventional PARAFAC2 and probabilistic PARAFAC2 models fitted with both the true number of components ((**a**,**b**), with $M=\tilde{M}=4$) and with an overspecified number of components ((**c**,**d**), with $\tilde{M}=6$). In the legend, Δ indicates a homoscedastic noise model and Ω indicates a heteroscedastic noise model.

The probabilistic PARAFAC2 models were fitted to the data sets with the results on the synthetic data shown in Figure 1 and Figure 2. To investigate the effect of the principled model selection approach based on the ELBO, we compare it to the existing model order selection heuristics by plotting the different selection criteria as a function of the number of components used in the model in Figure 1a,b. The figures show the mean result of the models fitted on the 10 synthetic data sets with four components and an SNR of 4. Overall, the ELBO suggests the same number of components as the other two criteria, R2 and CCD. When the data have heteroscedastic noise, the two probabilistic models that incorporate this have a substantially higher ELBO compared to the homoscedastic models.

The results for varying SNR using the true number of components in each model are shown in Figure 2a for data with homoscedastic noise and in Figure 2b for data with heteroscedastic noise. We report the R2 on the noiseless data, i.e., using the formula from (5), with the modification that the noise $E_{k}$ has been subtracted from $X_{k}$ for each slab. Thereby, we measure the different models’ ability to capture the true underlying structure in the data.

On the homoscedastic data, we see a small advantage of using the two vMF models compared to the direct fitting algorithm when we decrease the SNR of the data. The cMN model performs slightly worse compared to the direct fitting algorithm. When we move to the heteroscedastic data, we see a stronger separation of the four different probabilistic methods. Naturally, the models with heteroscedastic noise outperform the ones with homoscedastic noise. It is also evident that the penalty of modeling the noise as heteroscedastic in a setting where the true noise is homoscedastic is small.

If the number of components is misspecified, see Figure 2c,d, we see a larger difference between the performance of the probabilistic models accounting for the heteroscedastic noise and the direct fitting algorithm. Here, we also observe that the vMF models perform better compared to the cMN parameterization and see a larger positive effect of using the probabilistic models over the direct fitting algorithm. This is mainly explained by the reduced tendency to overfit when accounting for the uncertainty and the automatic relevance determination (ARD) pruning irrelevant components, as the Bayesian modeling promotes simpler representations by the ARD.

### 3.2. Real Data

As our synthetic results suggest, both formulations of the orthogonality constraint appear to be reasonable; we further investigate their performance on three real-world data sets. The first is an amino acid fluorescence (AAF) data set (available at www.ucphchemometrics.com (accessed on 28 February 2017), previously http://www.models.life.ku.dk/Amino_Acid_fluo) described in [61,66], in which the core-consistency diagnostic based on the PARAFAC2 model has previously successfully identified the three underlying constituents; tyrosine, tryptophan, and phenylalanine [54]. The data set contains five samples with 201 emission and 61 excitation intervals.

In addition, the models were evaluated on two gas chromatography–mass spectrometry (GC-MS) data sets. The first of these originated from wine (GC-MS-WINE) (available at www.ucphchemometrics.com (accessed on 28 February 2017), previously http://www.models.life.ku.dk/Wine_GCMS_FTIR) and was described in detail in [67]. PARAFAC2 has previously been used on GC-MS data obtained from measuring wine [54,68]. The second data set based on tobacco (GC-MS-TOBAC) was produced by [69] and kindly made available by the authors upon request. The GC-MS-WINE data contain 44 samples of wine; here, we specifically consider the unaligned data at the elution times 4.5903–4.7527 min over the mass range *m*/*z* 5–204. The GC-MS-TOBAC data analyzed here contain 65 samples of tobacco, and we consider the elution times between 4.95 and 5.03 min over the mass range *m*/*z* 50–350.

**Figure 3:**
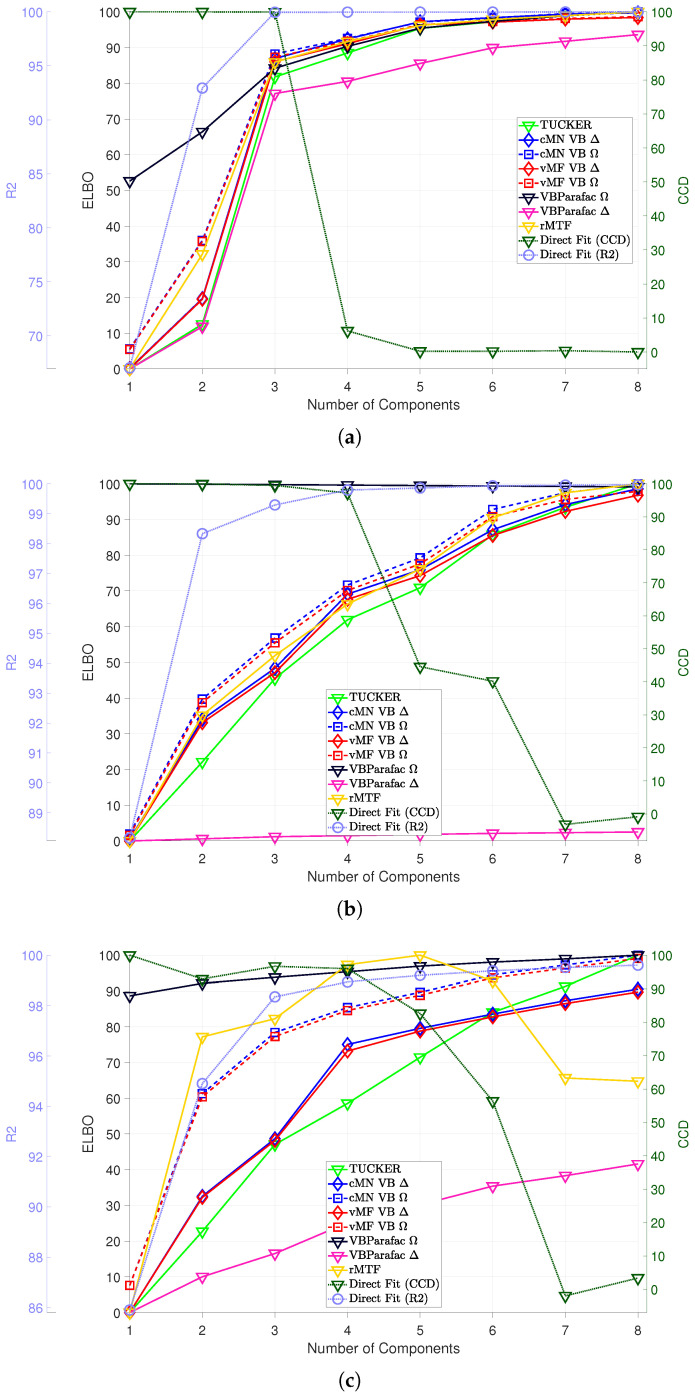
Mean of model selection criteria R2 and CCD reported on the conventional PARAFAC2, and the ELBO for the TUCKER, rMTF, probabilistic PARAFAC, and probabilistic PARAFAC2 models with 1 to 8 components on the AAF (**a**), GC-MS-WINE (**b**), and GC-MS-TOBAC (**c**) data sets. To make the results comparable, all ELBO values for each criterion and model (but across noise model types) have been normalized to be in the range of 0 to 100. In the legend, Δ indicates a homoscedastic noise model and Ω indicates a heteroscedastic noise model.

**Figure 4:**
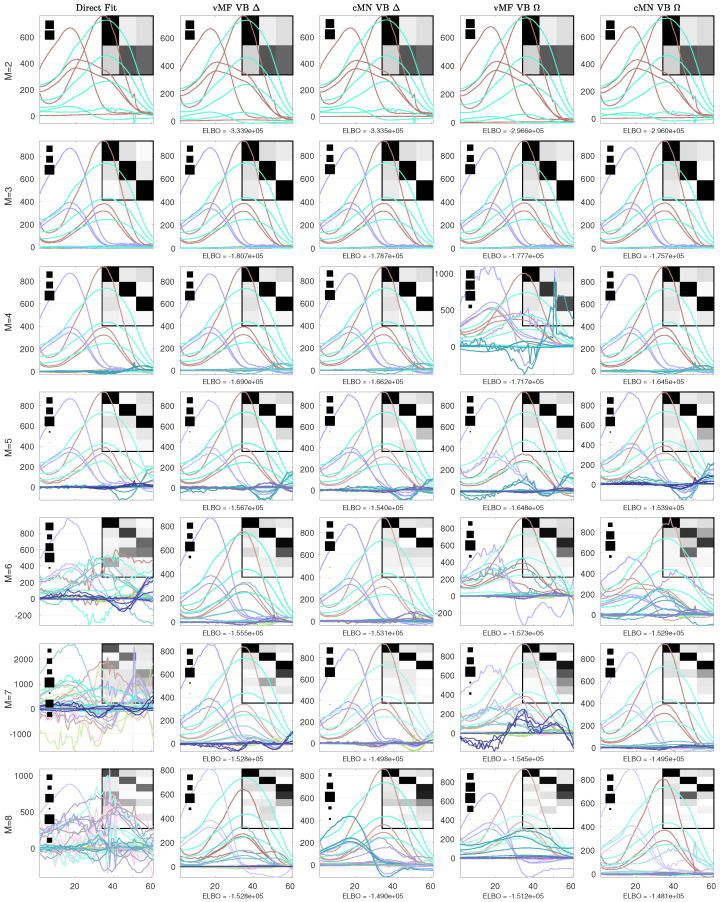
The excitation loadings of the AAF data given by the conventional PARAFAC2 and probabilistic PARAFAC2 models. From top to bottom, the loadings consist of 2 to 8 components. For each model, the background heatmap visualizes the correlation between the data reconstruction for each identified component and the componentwise data reconstruction of the conventional PARAFAC2 model with 3 components (ground-truth). Furthermore, to the left, a Hinton diagram indicates the relative squared Frobenius norm of the componentwise data reconstructions to the sum of them all. In the headers, Δ indicates a homoscedastic noise model and Ω indicates a heteroscedastic noise model.

**Figure 5:**
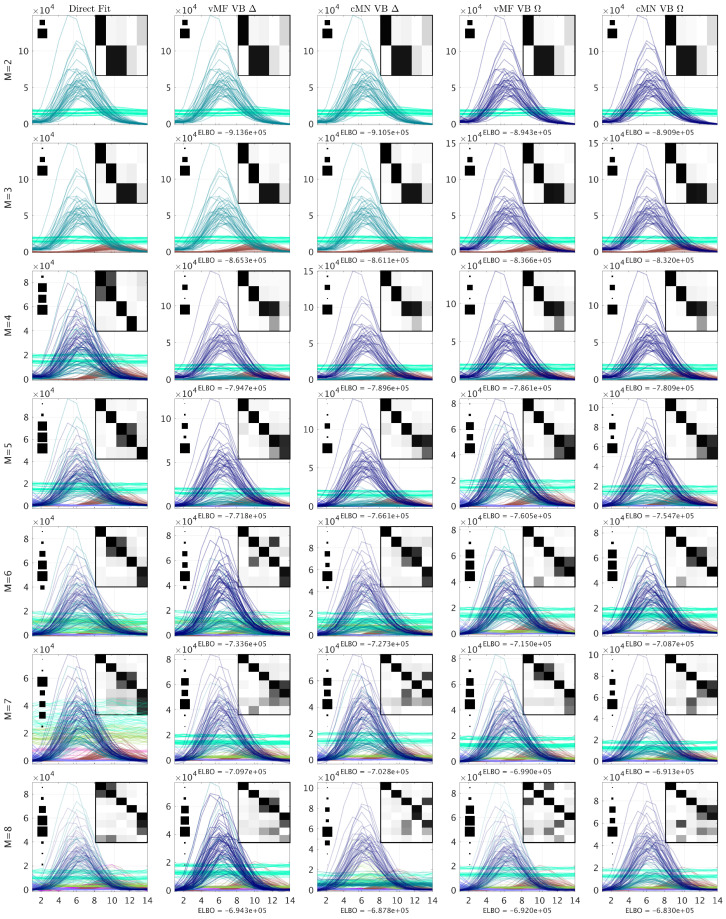
The elution profiles of the GC-MS-WINE data given by the conventional PARAFAC2 and probabilistic PARAFAC2 models. From top to bottom, the profiles consist of 2 to 8 components. For each model, the background heatmap visualizes the correlation between the data reconstruction for each identified component and the componentwise data reconstruction of the conventional PARAFAC2 model with 5 components (expert conclusion). Furthermore, to the left, a Hinton diagram indicates the relative squared Frobenius norm of the componentwise data reconstructions to the sum of them all. In the headers, Δ indicates a homoscedastic noise model and Ω indicates a heteroscedastic noise model.

**Figure 6:**
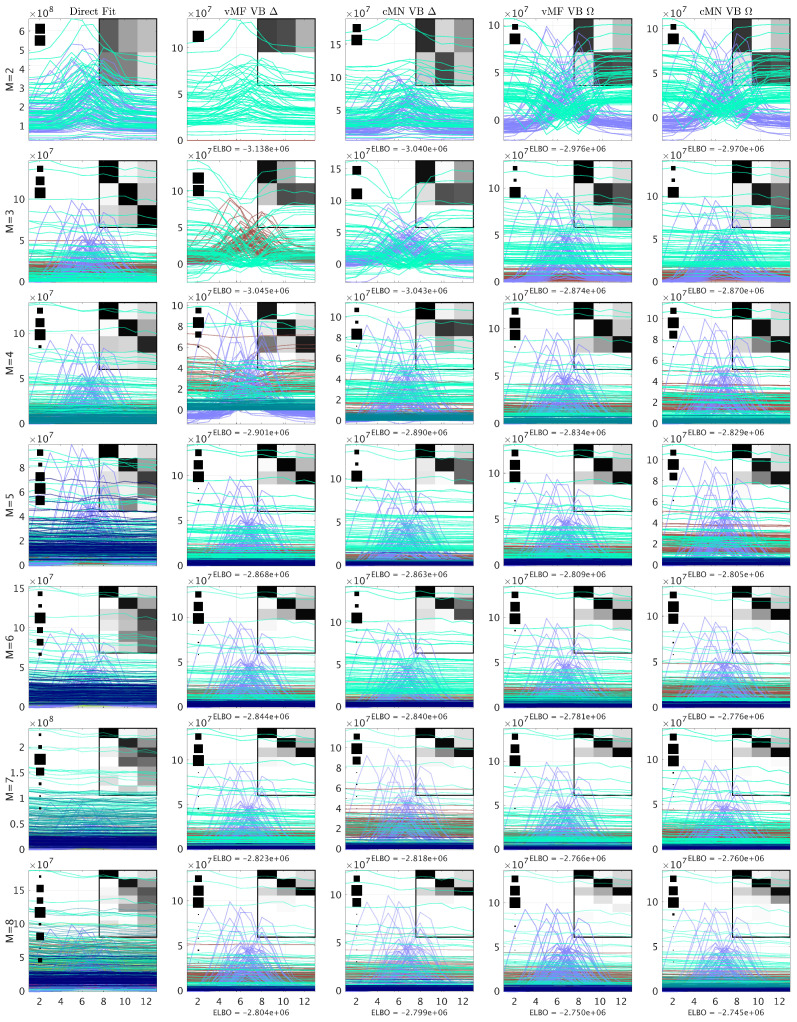
The elution profiles of the GC-MS-TOBAC data given by the conventional PARAFAC2 and probabilistic PARAFAC2 models. From top to bottom, the profiles consist of 2 to 8 components. For each model, the background heatmap visualizes the correlation between the data reconstruction for each identified component and the componentwise data reconstruction of the conventional PARAFAC2 model with 3 components (expert conclusion). Furthermore, to the left, a Hinton diagram indicates the relative squared Frobenius norm of the componentwise data reconstructions to the sum of them all. In the headers, Δ indicates a homoscedastic noise model and Ω indicates a heteroscedastic noise model.

In Figure 3, Figure 4, Figure 5 and Figure 6, we consider the estimated components using the direct fitting algorithm and the proposed probabilistic PARAFAC2 with homo- and heteroscedastic noise, respectively. In Figure 3, we report the ELBO using the probabilistic models as well as the R2 and CCD using the direct fitting algorithm, and in Figure 4, Figure 5 and Figure 6, we present the extracted profiles for each data set.

For the amino acid fluorescence data, we observe that both the R2 and CCD strongly suggest that a three-component model sufficiently describes the data, and the ELBO also finds no substantial improvements beyond three components (Figure 3a). In Figure 4, we investigate the extracted excitation loadings and observe that both the probabilistic and direct fitting PARAFAC2 models extract similar components when too few or the correct number of components are specified, i.e., M≤3. However, facing misspecification by having chosen too many components, the direct fitting algorithm extracts noisy profiles that incorrectly reflect the underlying three constituents. In contrast, the probabilistic PARAFAC2 models more robustly recover the three constituents when overspecifying the number of components—in particular, when assuming homoscedastic noise.

For the GC-MS-WINE data, the R2 and CCD point to a four- or five-component model, whereas the ELBO points to adding additional components (cf. Figure 3b). Inspecting the extracted components in Figure 5, we again observe close agreement between the extracted components using the probabilistic and direct fitting PARAFAC2 approaches when specifying a low number of components (M≤5). Furthermore, the estimated elution profiles facing model order misspecification appear less influenced by noise than the elution profiles extracted using the direct fitting algorithm, emphasizing the improved robustness by the Bayesian approach.

For the GC-MS-TOBAC data given in Figure 3c, we observe support for a three-component model according to R2 and CCD, whereas it is harder to decide a suitable model order based on the ELBO. The change in the ELBO from two to three components for the homoscedastic noise models suggests that local maxima have been identified. Inspecting the extracted components in Figure 6, it is also evident that local maxima have been reached for most of the probabilistic PARAFAC2 models with M<4. For M>3, most of the probabilistic models successfully recover the three components without using the extra components, where the direct fitting algorithm splits the three components into multiple components.

Of the three considered data sets, the ELBO itself does not strongly indicate an optimal number of components; however, most of the probabilistic models still manage to recover the underlying structure given by the ground-truth or expert conclusion in spite of being overspecified. This is in sharp contrast to MAP estimation, where overspecification typically leads to degenerate solutions. We attribute this to the regularization invoked by accounting for uncertainty and the automatic relevance determination promoting the pruning of excess components. The relative importance of each component can be observed from the Hinton diagrams in Figure 4, Figure 5 and Figure 6. Each square in the Hinton diagrams indicates the relative contribution of each component to the full data reconstruction, computed as the squared Frobenius norm of the componentwise data reconstruction divided by the sum of the squared Frobenius norms of all the componentwise data reconstructions.

## 4. Conclusions

We developed a fully probabilistic PARAFAC2 model and demonstrated how orthogonality can be imposed in the context of variational inference in two different ways: Firstly, using the von Mises–Fisher matrix distribution, assuming $E[Y^{⊤}Y]=I$, as proposed in the context of variational PCA in [59]. Using this distribution forces all the realizations of the given matrix parameter to be orthogonal. Secondly, using the constrained matrix normal distribution, assuming $E\left[Y^{⊤}\right]E\left[Y\right]=I$, in which the mean is constrained to the Stiefel manifold. This effectively results in a more flexible model as only the expectation of the realizations of the matrix are orthogonal and not the realizations themselves. For the latter approach, we presently derived a simple closed-form solution based on optimizing the lower bound.

Both probabilistic PARAFAC2 approaches were able to successfully recover the underlying signal in synthetic data when considering homoscedastic or heteroscedastic added noise. However, we found that the specification of orthogonality based on vMF was more robust to noise than the specification based on cMN. In particular, we observed substantial noise robustness in the probabilistic PARAFAC2 models when compared to the conventional direct fitting approach, both when the correct model order was specified and when overestimating the number of components.

On the AAF data, the probabilistic PARAFAC2 framework was able to correctly identify the underlying constituents and demonstrated improved robustness to model misspecification when compared to the conventional direct fitting algorithm. The ELBOs of the probabilistic models suggest a model order of three components similar to the CCD and R2 heuristics computed from the direct fitting estimations. For the two gas chromatography–mass spectrometry data sets, GC-MS-WINE and GC-MS-TOBAC, we also observed agreement between the probabilistic and direct fitting PARAFAC2 models but with more mixed results. The model order is not so clearly evident from the ELBO on these data sets. However, we see that the automatic relevance determination suppresses unnecessary components fairly well on both data sets, ensuring robustness to overspecification of the model, which otherwise leads to degenerate solutions when the direct fitting approach is used. A few results from the probabilistic PARAFAC2 did not match the results of the direct fitting approach. This can most likely be explained by encountering local maxima, since variational methods are known to suffer from issues of underestimating uncertainty and thereby becoming overly confident on estimated parameters.

We attribute the performance improvements of probabilistic PARAFAC2 over conventional PARAFAC2 to the casting of PARAFAC2 as a Bayesian model, which approximates the posterior distribution of the parameters—rather than a point estimate as conventional PARAFAC2. Additionally, Bayesian inference, in general, enjoys more robustness to noise and overspecification of the model [48]. The proposed probabilistic PARAFAC2 models form an important step in the direction of applying probabilistic approaches to more advanced tensor decomposition approaches and a new direction for handling orthogonality constraints in probabilistic modeling—in general, using the proposed constrained matrix normal distribution framework, which has a simple variational update. In particular, we anticipate that the orthogonality constraints within a probabilistic setting may also be useful for the Tucker decomposition, in which orthogonality is typically imposed [5]; the block-term decompositions [70], in which orthogonality may be beneficial to impose within each block as previously considered using the vMF [48]; or to improve identifiability within the CP decomposition by imposing orthogonality as implemented in the n-way toolbox (http://www.models.life.ku.dk/nwaytoolbox, accessed on 28 February 2017). PARAFAC2 is actively being advanced and employed for new applications, e.g., recently, the higher-order block term decomposition has been embedded with a PARAFAC2 structure [71].

## Figures and Tables

**Table 1:** Overview of all the variational factors and their updates. Note that $P={\left\{P_{k}\right\}}_{k=1,2,\ldots ,K}$ is the set of projection matrices and (SVD) indicates the expression is decomposed by singular value decomposition (SVD) to obtain $U_{k}S_{k}V_{k}$.

Variational Factor	Update
$q\left(A\right)∼\prod_{i}N\left(\mu_{a_{i\cdot }},\Sigma_{a_{i\cdot }}\right)$	$\Sigma_{a_{i\cdot }}={\left(I_{M}+\sum_{k}E\left[\tau_{k}\right]E\left[D_{k}F^{⊤}P_{k}^{⊤}P_{k}FD_{k}\right]\right)}^{-1}$
	$\mu_{a_{i\cdot }}=\Sigma_{a_{i\cdot }}\sum_{k}E\left[\tau_{k}\right]E\left[D_{k}F^{⊤}P_{k}^{⊤}x_{i\cdot k}^{⊤}\right]$
$q\left(C\right)∼\prod N\left(\mu_{c_{k\cdot }},\Sigma_{c_{k\cdot }}\right)$	$\Sigma_{c_{k\cdot }}={\left(\mathrm{diag}\left(\alpha \right)+E\left[\tau_{k}\right]E\left[F^{⊤}P_{k}^{⊤}P_{k}F\right]\circ E\left[A^{⊤}\;A\right]\right)}^{-1}$
	$\mu_{c_{k\cdot }}=\Sigma_{c_{k\cdot }}E\left[\tau_{k}\right]\mathrm{diag}\left(E\left[F^{⊤}\right]E\left[P_{k}^{⊤}\right]X_{k}^{⊤}E\left[A\right]\right)$
$q\left(F\right)∼\prod_{m}N\left(\mu_{f_{m\cdot }},\Sigma_{f_{m\cdot }}\right)$	$\Sigma_{f_{m\cdot }}={\left(\sum_{k}E\left[\tau_{k}\right]E\left[D_{k}A^{⊤}AD_{k}\right]E\left[p_{\cdot mk}^{⊤}p_{\cdot mk}\right]+I_{M}\right)}^{-1}$
	$\mu_{f_{m\cdot }}=\Sigma_{f_{m\cdot }}(\sum_{k}E\left[\tau_{k}\right]\{E\left[{\left(P_{k}^{⊤}\right)}_{m}\right]X_{k}^{⊤}E\left[A\right]E\left[D_{k}\right]$
	$\;-E\left[D_{k}A^{⊤}AD_{k}\right]\sum_{m^{\prime }\setminus m}E\left[p_{\cdot mk}^{⊤}p_{\cdot m^{\prime }k}\right]f_{m^{\prime }\cdot }^{⊤}\})$
$q\left(P\right)∼\prod_{k}\mathrm{vMF}\left(B_{P_{k}}\right)$	$B_{P_{k}}=E\left[\tau_{k}\right]E\left[F\right]E\left[D_{k}\right]E\left[A^{\;⊤}\right]X_{k}$
	$\begin{array}{c} E\left[P_{k}\right]=V_{k}\Psi U_{k}^{⊤},\;\mathrm{where}\;B_{P_{k}}=U_{k}S_{k}V_{k}^{⊤}\left(\mathrm{SVD}\right) \end{array}$
	(Ψ given by [59], Appendix A.2)
$q\left(P\right)∼\prod_{k}c\mathrm{MN}\left(M_{P_{k}},I_{J},\Sigma_{P_{k}}\right)$	$\Sigma_{P_{k}}={\left(E\left[FD_{k}A^{⊤}AD_{k}F^{⊤}\right]+I\right)}^{-1}$
	$\begin{array}{c} M_{P_{k}}=V_{k}U_{k}^{⊤},\mathrm{where}\;E\left[\tau_{k}\right]E\left[F\right]E\left[D_{k}\right]E\left[A^{\;⊤}\right]X_{k}=U_{k}S_{k}V_{k}^{⊤}\left(\mathrm{SVD}\right) \end{array}$
$q\left(\tau \right)∼\prod_{k}\mathrm{Gamma}\left(a_{\tau_{k}},b_{\tau_{k}}\right)$	$a_{\tau_{k}}=a_{\tau }+\frac{I\cdot J}{2}$
	$b_{\tau_{k}}=(b_{\tau }^{-1}\;+\;\frac{1}{2}\mathrm{Tr}\left(X_{k}X_{k}^{⊤}\right)\;+\;\frac{1}{2}E\left[\mathrm{Tr}\left(AD_{k}F^{⊤}P_{k}^{⊤}P_{k}FD_{k}A^{\;⊤}\right)\right]$
	$\;-E\left[\mathrm{Tr}\left(AD_{k}F^{⊤}P_{k}^{⊤}X_{k}^{⊤}\right)\right]{)}^{-1}$
$\underset{\alpha_{m}}{\arg\;\max}\;\mathrm{ELBO}\left(\alpha_{m}\right)$	$\alpha_{m}=K{\left(\sum_{k}E\left[c_{km}^{2}\right]\right)}^{-1}$

## Data Availability

No new data were created or analyzed in this study. Data sharing is not applicable to this article.

## Abbreviations

The following abbreviations are used in this manuscript:

PCA	principal component analysis
SNR	signal-to-noise ratio
SVD	singular value decomposition
ARD	automatic relevance determination
ELBO	evidence lower bound
CCD	core consistency diagnostic
KL divergence	Kullback–Leibler divergence
vMF	Von Mises–Fisher
CP	CandeComp/PARAFAC

## Appendix A. Software

A MATLAB implementation of the probabilistic PARAFAC2 model was used to run all experiments and generate the results in the paper. The source code is available on GitHub (https://github.com/philipjhj/VBParafac2, accessed on 28 February 2017), including a guide on setup and usage.

## Appendix B. Deriving the Variational Inference

In the following, we derive the most important expressions used to identify the update rules of the model parameters. Below is a overview of the used notation.

### Appendix B.1. The Evidence Lower Bound (ELBO)

An expansion of the ELBO is shown here:

$$\begin{array}{cc} \mathrm{ELBO}(q(\theta ))= & E[\log p(X,\theta )]-E[\log q(\theta )]\\ = & E[\log p(X,A,C,F,P,\tau ,\alpha )]-E[\log q(A,C,F,P,\tau ,\alpha )]\\ = & E[\log p(X|A,C,F,P,\tau )p(C|\alpha )p(F)p(P)p(\tau )]\\ \; & -E[\log q\left(A\right)q\left(C\right)q\left(F\right)q\left(P_{k}\right)q\left(\tau \right)]\\ = & E[\log p(X|A,C,F,P,\tau )]+E[\log p(A)]+E[\log p(C|\alpha )]\\ \; & +E[\log p(F)]+E[\log p(P)]+E[\log p(\tau )]\\ \; & -E[\log q(A)]-E[\log q(C)]-E[\log q(F)]-E[\log q(P)]\\ \; & -E[\log q(\tau )]\\ = & E[\log p(X|A,C,F,P,\tau )]+E[\log p(A)]+E[\log p(C|\alpha )]\\ \; & +E[\log p(F)]+E[\log p(P)]+E[\log p(\tau )]\\ \; & +h(q(A))+h(q(C))+h(q(F))+h(q(P))\\ \; & +h(q(\tau )) \end{array}$$

How to derive each of these terms is shown in the following.

### Appendix B.2. Standard Moment Matching

As the formulation of the probabilistic PARAFAC2 model consists of the multivariate normal and gamma distribution, we expand the logarithm of their general expressions below. This will serve as a reference for identifying the parameters of the variational distribution when reading the derivations of the update rules.

#### Appendix B.2.1. Multivariate Normal Distribution

Deriving the log of the probability density function of the multivariate normal distribution amounts to

$$\begin{array}{cc} f(x_{1},\ldots ,x_{k}) & =N\left(\left[x_{1},\ldots ,x_{k}\right];\mu_{X},\Sigma_{X}\right)=N\left(X;\mu_{X},\Sigma_{X}\right)\\ f(x_{1},\ldots ,x_{k}) & ={\left(2\pi \right)}^{-\frac{k}{2}}\left(\right|\Sigma_{X}{\left|\right)}^{-\frac{1}{2}}\exp\left(-\frac{1}{2}{\left(X-\mu_{X}\right)}^{⊤}\Sigma_{X}^{-1}\left(X-\mu_{X}\right)\right)\\ \Rightarrow \ln f(x_{1},\ldots ,x_{k}) & =\ln\left[{\left(2\pi \right)}^{-\frac{k}{2}}\left(\right|\Sigma_{X}{\left|\right)}^{-\frac{1}{2}}\exp\left(-\frac{1}{2}{\left(X-\mu_{X}\right)}^{⊤}\Sigma_{X}^{-1}\left(X-\mu_{X}\right)\right)\right]\\ \; & =-\frac{k}{2}\ln\left(2\pi \right)-\frac{1}{2}\ln(|\Sigma_{X}\left|\right)-\frac{1}{2}{\left(X-\mu_{X}\right)}^{⊤}\Sigma_{X}^{-1}\left(X-\mu_{X}\right)\\ \; & =-\frac{k}{2}\ln\left(2\pi \right)-\frac{1}{2}\ln(|\Sigma_{X}\left|\right)-\frac{1}{2}X^{⊤}\Sigma_{X}^{-1}X-\frac{1}{2}{\mu_{X}}^{⊤}\Sigma_{X}^{-1}\mu_{X}+{\mu_{X}}^{⊤}\Sigma_{X}^{-1}X\\ \; & =-\frac{1}{2}\ln(|\Sigma_{X}\left|\right)-\frac{1}{2}X^{⊤}\Sigma_{X}^{-1}X-\frac{1}{2}{\mu_{X}}^{⊤}\Sigma_{X}^{-1}\mu_{X}+{\mu_{X}}^{⊤}\Sigma_{X}^{-1}X+c \end{array}$$

where *c* is the constant terms with respect to $X_{k}$ and its parameters.

#### Appendix B.2.2. Gamma Distribution

Deriving the log density function of the gamma distribution amounts to

$$\begin{array}{cc} f(x;a,b)= & \frac{1}{\Gamma \left(a\right)b^{a}}x^{a-1}\exp\left(-xb^{-1}\right)\\ \Rightarrow \ln f(x;a,b)= & \ln\left[\frac{1}{\Gamma \left(a\right)b^{a}}x^{a-1}\exp\left(-xb^{-1}\right)\right]\\ = & \ln\frac{1}{\Gamma \left(a\right)b^{a}}+\left(a-1\right)\ln x-xb^{-1}\\ = & \left(a-1\right)\ln x-xb^{-1}+c \end{array}$$

where *c* is the constant terms with respect to *x*.

### Appendix B.3. Non-Trivial Moment Matching

To identify the parameters for C and F, non-trivial steps had to be performed.

#### Appendix B.3.1. The F Matrix

The variational factor for F is defined as

$$\begin{array}{cc} q(F)\propto & \exp E_{-F}\left[\log p\left(X,\theta \right)\right]\\ \propto & \exp E_{-F}\left[\log p\left(X,F|A,C,P,\tau \right)\right] \end{array}$$

where

$$\begin{array}{cc} E_{-F}\left[\log p\left(X,F|A,C,P,\tau \right)\right]= & E_{-F}\left[\log p\left(X|A,C,F,P,\tau \right)\right]+E_{-F}\left[\log p\left(F\right)\right]\\ = & \sum_{k}\sum_{i}E_{-F}\left[\log p\left(x_{i\cdot k}|a_{i\cdot },D_{k},F,P_{k},\tau_{k}\right)\right]+\sum_{m}E_{-F}\left[\log p\left(f_{m\cdot }\right)\right]\\ = & \sum_{k}\sum_{i}E_{-F}[-\frac{1}{2}\left(a_{i\cdot }D_{k}F^{⊤}P_{k}^{⊤}\right)I_{M}\tau_{k}{\left(a_{i\cdot }D_{k}F^{⊤}P_{k}^{⊤}\right)}^{⊤}\\ \; & +a_{i\cdot }D_{k}F^{⊤}{P_{k}}^{⊤}I_{M}\tau_{k}{x_{i\cdot k}}^{⊤}]+\sum_{m}E_{-F}\left[-\frac{1}{2}f_{m\cdot }I_{M}{f_{m\cdot }}^{⊤}\right]+c\\ = & -\frac{1}{2}\sum_{k}\sum_{i}E_{-F}\left[\tau_{k}\left(a_{i\cdot }D_{k}F^{⊤}{P_{k}}^{⊤}P_{k}FD_{k}{a_{i\cdot }}^{⊤}\right)\right]-\frac{1}{2}\sum_{m}f_{m\cdot }{f_{m\cdot }}^{⊤}\\ \; & +\sum_{k}\sum_{i}E_{-F}\left[\tau_{k}a_{i\cdot }D_{k}F^{⊤}{P_{k}}^{⊤}{x_{i\cdot k}}^{⊤}\right]+c\\ = & -\frac{1}{2}\sum_{k}E\left[\tau_{k}\right]\sum_{i}E_{-F}\left[a_{i\cdot }D_{k}F^{⊤}{P_{k}}^{⊤}P_{k}FD_{k}{a_{i\cdot }}^{⊤}\right]-\frac{1}{2}\sum_{m}f_{m\cdot }{f_{m\cdot }}^{⊤}\\ \; & +\sum_{k}\sum_{i}E\left[\tau_{k}\right]E_{-F}\left[a_{i\cdot }D_{k}F^{⊤}{P_{k}}^{⊤}{x_{i\cdot k}}^{⊤}\right]+c. \end{array}$$

Again, we reorder the parameters using the trace operator to identify the quadratic term. This time, the quadratic term separates into a quadratic and linear part revealing a linear intercomponent dependency.

$$\begin{array}{cc} E_{-F}\left[a_{i\cdot }D_{k}F^{⊤}{P_{k}}^{⊤}P_{k}FD_{k}{a_{i\cdot }}^{⊤}\right]= & E_{-F}\left[\mathrm{Tr}\left(a_{i\cdot }D_{k}F^{⊤}{P_{k}}^{⊤}P_{k}FD_{k}{a_{i\cdot }}^{⊤}\right)\right]\\ = & E_{-F}\left[\mathrm{Tr}\left(FD_{k}{a_{i\cdot }}^{⊤}a_{i\cdot }D_{k}F^{⊤}{P_{k}}^{⊤}P_{k}\right)\right]\\ = & \mathrm{Tr}(FE_{-F}\left[D_{k}{a_{i\cdot }}^{⊤}a_{i\cdot }D_{k}\right]F^{⊤}E_{-F}\left[{P_{k}}^{⊤}P_{k}\right])\\ = & \sum_{mm^{\prime }}{\left(FE\left[D_{k}{a_{i\cdot }}^{⊤}a_{i\cdot }D_{k}\right]F^{⊤}\right)}_{mm^{\prime }}{\left(E\left[{P_{k}}^{⊤}P_{k}\right]\right)}_{mm^{\prime }}\\ = & \sum_{mm^{\prime }}f_{m\cdot }E\left[D_{k}{a_{i\cdot }}^{⊤}a_{i\cdot }D_{k}\right]f_{m^{\prime }\cdot }^{T}E\left[p_{\cdot mk}^{T}p_{\cdot m^{\prime }k}\right]\\ = & \sum_{m}f_{m\cdot }E\left[D_{k}{a_{i\cdot }}^{⊤}a_{i\cdot }D_{k}\right]E\left[p_{\cdot mk}^{T}p_{\cdot mk}\right]f_{m\cdot }^{T}\\ \; & +2\sum_{m}\sum_{m^{\prime }\setminus m}f_{m\cdot }E\left[D_{k}{a_{i\cdot }}^{⊤}a_{i\cdot }D_{k}\right]E\left[p_{\cdot mk}^{T}p_{\cdot m^{\prime }k}\right]f_{m^{\prime }\cdot }^{T} \end{array}$$

Again, we have to reorder and include the linear terms as before.

$$\begin{array}{cc} \sum_{i}E_{-F}\left[a_{i\cdot }D_{k}F^{⊤}{P_{k}}^{⊤}{x_{i\cdot k}}^{⊤}\right]= & \sum_{i}\sum_{m}E_{-F}\left[a_{i\cdot }D_{k}{f_{m\cdot }}^{⊤}{\left({P_{k}}^{⊤}\right)}_{m}{x_{i\cdot k}}^{⊤}\right]\\ = & \sum_{i}\sum_{m}E\left[a_{i\cdot }\right]E\left[D_{k}\right]{f_{m\cdot }}^{⊤}E\left[{\left({P_{k}}^{⊤}\right)}_{m}\right]{x_{i\cdot k}}^{⊤}\\ = & \sum_{i}\sum_{m}E\left[{\left({P_{k}}^{⊤}\right)}_{m}\right]{x_{i\cdot k}}^{⊤}E\left[a_{i\cdot }\right]E\left[D_{k}\right]{f_{m\cdot }}^{⊤}\\ = & \sum_{m}E\left[{\left({P_{k}}^{⊤}\right)}_{m}\right]\left(\sum_{i}{x_{i\cdot k}}^{⊤}E\left[a_{i\cdot }\right]\right)E\left[D_{k}\right]{f_{m\cdot }}^{⊤} \end{array}$$

Accounting for all terms and matching them to the ones in Appendix B.2, we arrive at the following update rules for F.

(A1) $$q\left(F\right)=\prod_{m}N\left(\mu_{f_{m\cdot }},\Sigma_{f_{m\cdot }}\right),$$

(A2) $$\mu_{f_{m\cdot }}=\Sigma_{f_{m\cdot }}\left(\sum_{k}E\left[\tau_{k}\right]\left(E\left[{\left({P_{k}}^{⊤}\right)}_{m}\right]{X_{k}}^{⊤}E\left[A\right]E\left[D_{k}\right]-\sum_{i}E\left[D_{k}{a_{i\cdot }}^{⊤}a_{i\cdot }D_{k}\right]\sum_{m^{\prime }\setminus m}E\left[p_{\cdot mk}^{T}p_{\cdot m^{\prime }k}\right]f_{m^{\prime }\cdot }^{T}\right)\right),$$

(A3) $$\Sigma_{f_{m\cdot }}={\left(\sum_{k}E\left[\tau_{k}\right]\sum_{i}E\left[D_{k}{a_{i\cdot }}^{⊤}a_{i\cdot }D_{k}\right]E\left[p_{\cdot mk}^{T}p_{\cdot m^{\prime }k}\right]+I_{M}\right)}^{-1}.$$

#### Appendix B.3.2. Constrained Matrix Normal Distribution

The orthogonality constraint in the model can be handled with two formulations. This section concerns the approach where the mean parameters of the variational approximation for $P_{k}$ are constrained to be orthogonal, and the following section describes the solution using the von Mises–Fisher distribution. Instead of using the free form variational updates, we optimized the ELBO with respect to the mean parameters $M_{P_{k}}=E\left[P_{k}\right]$ constrained to be orthogonal.

$$\begin{array}{ccc} M_{P_{k}}= & \underset{M_{P_{k}}}{\arg\;\max}\;\mathrm{ELBO}\left(M_{P_{k}}\right) & s.t.\;M_{P_{k}}{M_{P_{k}}}^{⊤}=I\\ = & \underset{M_{P_{k}}}{\arg\;\max}\;E\left[\log p\left(X|A,C,F,P,\tau \right)\right]\\ \; & +E[\log p(A)]+E[\log p(C|\alpha )]\\ \; & +E[\log p(\alpha )]+E[\log p(F)]+E[\log p(P)]+E[\log p(\tau )]\\ \; & +h(q(A))+h(q(C))+h(q(F))+h(q(P))\\ \; & +h(q(\tau ))+h(q(\alpha )) & s.t.\;M_{P_{k}}{M_{P_{k}}}^{⊤}=I\\ = & \underset{M_{P_{k}}}{\arg\;\max}\;E\left[\log p\left(X|A,C,F,P,\tau \right)\right]+c_{1} & s.t.\;M_{P_{k}}{M_{P_{k}}}^{⊤}=I\\ = & \underset{M_{P_{k}}}{\arg\;\max}\;-\frac{1}{2}\sum_{k}\sum_{i}E\left[\tau_{k}\left(a_{i\cdot }D_{k}F^{⊤}{P_{k}}^{⊤}P_{k}FD_{k}{a_{i\cdot }}^{⊤}\right)\right]\\ \; & +\sum_{k}\sum_{i}E\left[\tau_{k}a_{i\cdot }D_{k}F^{⊤}{P_{k}}^{⊤}{x_{i\cdot k}}^{⊤}\right]+c_{2}\; & s.t.\;M_{P_{k}}{M_{P_{k}}}^{⊤}=I\\ = & \underset{M_{P_{k}}}{\arg\;\max}\;\sum_{k}\sum_{i}E\left[\tau_{k}a_{i\cdot }D_{k}F^{⊤}{P_{k}}^{⊤}{x_{i\cdot k}}^{⊤}\right]+c_{3}\; & s.t.\;M_{P_{k}}{M_{P_{k}}}^{⊤}=I\\ = & \underset{M_{P_{k}}}{\arg\;\max}\;\sum_{k}E\left[\tau_{k}\right]\mathrm{Tr}\left(E\left[A\right]E\left[D_{k}\right]E\left[F^{⊤}\right]E\left[{P_{k}}^{⊤}\right]{X_{k}}^{⊤}\right)+c_{3}\; & s.t.\;M_{P_{k}}{M_{P_{k}}}^{⊤}=I\\ = & \underset{M_{P_{k}}}{\arg\;\max}\;\sum_{k}E\left[\tau_{k}\right]\mathrm{Tr}\left(E\left[F\right]E\left[D_{k}\right]E\left[A^{⊤}\right]X_{k}M_{P_{k}}\right)+c_{3}\; & s.t.\;M_{P_{k}}{M_{P_{k}}}^{⊤}=I. \end{array}$$

Only the linear term of the probability density function of the data X depends on $M_{P_{k}}$ since $M_{P_{k}}$ in the quadratic terms is the identity matrix. Except for a scalar, the optimization problem reduces to the same one as finding $P_{k}$ in the alternating least squares algorithm, where one maximizes $\mathrm{Tr}(E\left[F\right]E\left[D_{k}\right]E\left[A^{⊤}\right]X_{k}M_{P_{k}})$ subject to the orthogonality constraint. The solution to this is found by simply applying an SVD, as stated in the main text (The alternating least squares method is described in [15], and the solution to the optimization problem was first described in [52]).
